# Biomimicking Covert Communication by Time-Frequency Shift Modulation for Increasing Mimicking and BER Performances

**DOI:** 10.3390/s21062184

**Published:** 2021-03-20

**Authors:** Jongmin Ahn, Hojun Lee, Yongcheol Kim, Wanjin Kim, Jaehak Chung

**Affiliations:** 1 Department of Electronics Engineering, INHA University, Inhceon 22201, Korea; anjong3@naver.com (J.A.); timmit@naver.com (H.L.); dydcjf4691@naver.com (Y.K.); 2 Agency of Defense Development, Changwon-si 51682, Korea; kimwj@add.re.kr

**Keywords:** convert communication, bio-mimetic, signal processing, underwater communication, MOS test

## Abstract

Underwater acoustic (UWA) biomimicking communications have been developed for covert communications. For the UWA covert communications, it is difficult to achieve the bit error rate (BER) and the degree of mimic (DoM) performances at the same time. This paper proposes a biomimicking covert communication method to increase both BER and DoM (degree of mimic) performances based on the Time Frequency Shift Keying (TFSK). To increase DoM and BER performances, the orthogonality requirements of the time- and frequency-shifting units of the TFSK are theoretically derived, and the whistles are multiplied by the sequence with a large correlation. Two-step DoM assessments are also developed for the long-term whistle signals. Computer simulations and practical lake and ocean experiments demonstrate that the proposed method increases the DoM by 35% and attains a zero BER at −6 dB of Signal to Noise Ratio (SNR).

## 1. Introduction

Underwater acoustic (UWA) covert communication requires covertness and confidentiality. The conventional UWA covert communication schemes achieve the covertness using the spread spectrum that spreads out the communication signal over a wide frequency band, which is considered as background noise [1,2,3,4,5,6,7]. However, the narrow bandwidth of the UWA communication cannot utilize a large spreading factor, and a low transmit power for the covertness causes a short available communication range [1,2,3,4,5,6,7]. As an alternate, biomimetic communications have been developed, which mimic the dolphin whistles to increase the covertness [8,9,10,11,12,13]. The idea of biomimetic communications is to make the enemy confuse the communication signals with dolphin sounds [8,9,10,11,12,13,14].

The CV-CFM (continuously varying-carrier frequency modulation) method was developed for biomimetic communication [12]. The CV-CFM selects one whistle among many whistles, and divides the whistle into many short-time periods, and modulates binary bits to the periods using the conventional digital communication modulations (i.e., Chirp Spread Spectrum (CSS), Frequency Shift Keying (FSK), and Phase Shift Keying (PSK)) [9,10,12]. The short division of the whistle increased the frequency bandwidth of the whistle pattern, which decreased the degree of mimic (DoM) and Bit Error Ratio (BER) performances. To increase the DoM and BER performances, the time-frequency shift keying (TFSK) method was researched [13]. The TFSK method did not allocate the binary information bits to the divided whistles, but to the shifted locations in the time-frequency of the whistles [13]. However, the BER performance of the TFSK varied with the transmitted whistle patterns, and the time- and frequency-shifting units that satisfy the orthogonality in the time- and frequency-domains were not derived. If a few whistle patterns with a low BER are used for a long data sequence, the DoM of Ref. [13] decreases by the repeated similar whistles. For the practical biomimetic covert communication, the large DoM and the low BER need to be simultaneously achieved and to be evaluated according to various parameters of the real dolphin sounds, e.g., long-term signal duration, bandwidth, etc. [8,9,10,11,12,13,14]. In general, the time duration and the frequency bandwidth of the dolphin whistles vary from several hundred milliseconds to two seconds and from several hundred Hz to tens of kHz, respectively [15,16,17,18,19]. Thus, the DoM evaluation needs to be performed for a long-term period.

This paper proposes a TFSK-based biomimetic communication method increasing the DoM and BER performances. We theoretically derive the orthogonality requirements in the time- and frequency-domains and utilizes various whistle patterns with sequences with the large autocorrelation to increases the DoM and BER performances. To evaluate the long-term DoM of the proposed method, a two-step DoM assessment is developed. The computer simulation and the ocean experiments were executed to show that the proposed method demonstrated better DoM and BER performances than the conventional convert communications.

The main contributions of the paper are summarized as follows:

1. The time- and frequency-shifting unit requirements of the TFSK are theoretically derived for the orthogonality in the time- and frequency-domains. The requirements guarantee the low BER.
2. For the large DoM and the low BER, the sequence with a large correlation is multiplied to the whistles, which enables to use of various dolphin whistles without any restrictions. In addition, the orthogonality requirements for the proposed method are also derived.
3. Since the sequence makes the whistle spread in the frequency domain, the DoM assessments are conducted to find the unrecognizable spreading parameter. Thus, a two-step DoM assessment is proposed: The 1st step is conducted to find the best length of the sequence. The 2nd step is executed to confirm whether the long-term whistles signal with the selected sequence length is acceptable for the covert communication.
4. The computer simulations and the practical lake and ocean experiments were conducted and demonstrated the proposed method had the large DoM and the lower BER compared with the conventional covert communication methods.

This paper is organized as follows. In Section 2, the orthogonal requirement of the TFSK according to a whistle pattern is derived, and the proposed biomimetic communication method is described. Section 3 explains the DoM assessment method for the proposed biomimetic communication signals. In Section 4, the BER and DoM performances of the proposed method are analyzed. In Section 5, the proposed biomimetic communication method demonstrated the large DoM and the low BER through computer simulations and practical lake and ocean experiments. Section 6 concludes the paper.

## 2. Proposed Method

**Figure 1:**
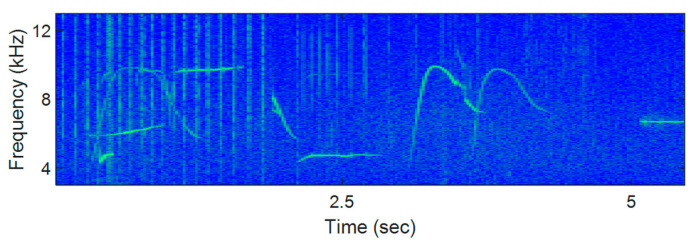
Spectrograms of the various real Delphinus delphis whistles.

The conventional TFSK scheme modulates binary bits to the shifted time-frequency position of the whistles [13]. Ref. [13] does not provide the requirements of the time- and frequency-shifting units which guarantee the orthogonality of whistles, and if zero-chirp rate whistles are transmitted, the detection performance degrades by the time ambiguity. Thus, few selected whistles may be utilized to attain the large BER. Figure 1 shows the spectrograms of the real whistles. In Figure 1, the zero-chirp rate whistles are frequently found. If these zero-chirp rate whistles are not transmitted to avoid the detection ambiguity of the TFSK, the DoM of the TFSK decreases for the long-term observation.

We theoretically derive the orthogonality requirements in time- and frequency-shifting units and propose a novel scheme that multiplies the sequence with the large autocorrelation to the whistles to increase the BER performance in time. Thus, all whistles are utilized for the transmission whistles, which increases the DoM.

### 2.1. TFSK Performance Analysis According to Whistle Pattern

For the derivation of the time- and frequency-shifting unit requirements, the whistles are mathematically modeled, and the orthogonality requirements are derived. Assume that the whistle pattern is modeled as a function ($f_{w}\left(t\right)$), which is presented as [12]:

(1) $$w\left(t\right)=cos\left[\int f_{w}\left(t\right)dt\right].$$

Since the conventional TFSK modulation scheme shifts the whistle patterns by the time and the frequency according to transmit the binary bits, let the time- and frequency-shifting units be Δt and Δf, respectively, and the total number of time and frequency grids be M and N, respectively. If the whistle is modulated with input bits, a reference whistle is shifted from $-\Delta t\frac{M-1}{2}$ to $\Delta t\frac{M-1}{2}$ in the time domain and from $-\Delta f\frac{N-1}{2}$ to $\Delta f\frac{N-1}{2}$ in the frequency domain, respectively. Gray coding to the time-frequency mapping may be utilized to increase the BER. For M and N grids, the TFSK modulation conveys $\left(\;log_{2}\left(N\right)+log_{2}\left(M\right)\right)$ bits per one whistle.

**Figure 2:**
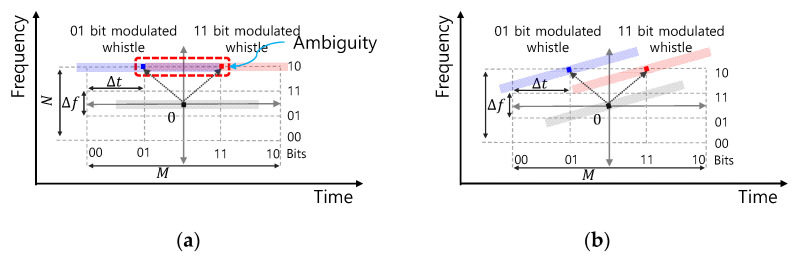
Examples of Time Frequency Shift Keying (TFSK) modulated signals: (**a**) zero-chirp rate whistle pattern (chirp rate: 0 Hz/s), (**b**) Up-chirp whistle pattern (chirp rate is larger than 0 Hz/s).

If the whistle is modulated with m and n grid, which is an integer less than M and M, respectively, the TFSK modulated whistle ($S_{TF}\left(t\right)$) is expressed as:

(2) $$S_{TF}\left(t\right)=\left[\delta \left(t-m\Delta t\right)⊗w\left(t\right)\right]\times e^{j2\pi n\Delta ft},$$

where ⊗ denotes a convolutional operation. Figure 2 shows the two different TFSK modulated whistle examples.

In Figure 2, M and N are four each and a total of four bits are allocated. The gray-colored whistle denotes a reference whistle, and blue and red whistles denote the TFSK modulated ones. In Figure 2a, a zero-chirp rate whistle is modulated. The red and blue whistles are modulated by 01 and 11 in time, respectively, and by 10 in frequency. In Figure 2b, a whistle with a large-chirp rate is modulated. The red and blue ones are modulated by 01 and 11 in time, respectively, and by 10 in frequency. In Figure 2a, the blue and the red whistles are overlapped in the time-frequency domain because of the zero-chirp rate whistle and the small Δt, and the receiver is unable to correctly determine the transmitted bits by the overlapped region. If Δt is larger than that of the whistle length, two whistles are separated in the time domain and the detection of the whistle has no ambiguity. Then, the receiver correctly decodes the transmitted bits. In Figure 2b, however, even though the same `Δt is utilized, two modulated whistles are orthogonal in the time-frequency domain, and the receiver has a low BER. Thus, the BER performance of the TFSK method depends on the whistle pattern, and to increase the BER, finding the good Δt and Δf satisfying the orthogonal requirements of the TFSK needs for the given whistle pattern.

For Δf, the calculation of the orthogonality requirement for an arbitrary whistle pattern is difficult. However, if the receiver demodulates the received signal using the multiplication of the complex conjugate to the transmitted whistle, the demodulated signal has a zero-chirp rate pattern. Then, the orthogonality requirement of Δf becomes similar to that of the conventional FSK modulation. When the time length of the whistle is $L_{w}$, the Δf satisfying orthogonal requirements of the TFSK is given as:

(3) $$\Delta f>1/L_{w}.$$

For Δt, more manipulation is needed. Firstly, assume that Δt is less than $L_{w}$. If Δt is larger than $L_{w}$, no overlap and no misdetection occur, but the data rate decreases. Thus, this case is not considered in this paper. Since the whistle patterns are varied and non-linear function, the derivation of the orthogonality requirement of Δt for the whistles is difficult. If the non-linear whistle pattern is divided into short-intervals, a piece of the whistle can simply be modeled as a linear function ($f_{w}\left(t\right)$), e.g., 1$f_{w}\left(t\right)=at+b$ with a chirp rate (a), which is defined as a=df/Δt where Δt and df denote the time- and frequency-differences, respectively. Thus, if Δt is derived from the smallest chirp-rate of the whistle, the derived Δt satisfies the orthogonality of the whistle with the length ($L_{w}$).

**Figure 3:**
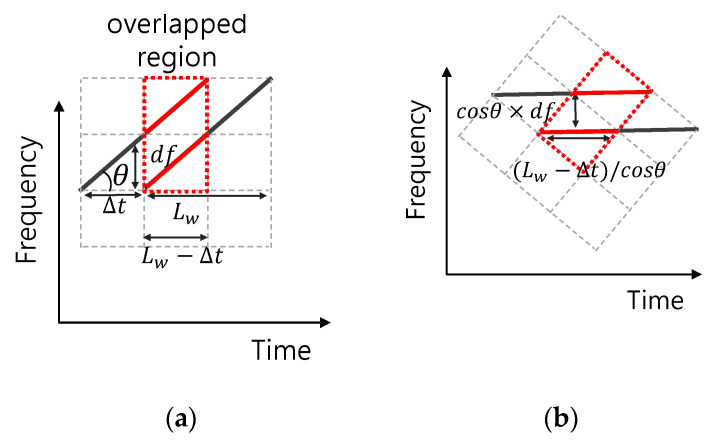
TFSK modulation whistle; (**a**) Normal, (**b**) Rotation by θ.

Assume that θ is the decline of the whistle in the time-frequency domain and defined as $\theta =tan^{-1}\left(df/\Delta t\right)$. To calculate the orthogonality requirement of the whistle with a, we rotate two whistles to θ clockwise in the time-frequency domain in Figure 3. The two modulated whistles are separated in the time-frequency domain keeping the orthogonality. In Figure 3a, if one whistle is shifted by Δt in the time domain, the overlapped time is given as $\left(L_{w}-\Delta t\right)$ and the frequency difference is given as df Hz. In Figure 3b, after the rotation by θ, the time duration of $\left(L_{w}-\Delta t\right)/cos\;\theta$ is overlapped in the time domain and the frequency gap of cosθ×df is obtained in the frequency domain.

If the frequency gap in Figure 3b is larger than the inverse of the overlapped time duration, two whistles satisfy the orthogonality in the time-frequency domain. Therefore, the orthogonality requirement for Δt is calculated as:

(4) $$cos\theta \times df\geq cos\theta /\left(L_{w}-\Delta t\right),0<\Delta t<L_{w}.$$

Since df is equal to aΔt in Figure 3a, Equation (4) is rewritten as:

(5) $$a\geq 1/\left((L_{w}-\Delta t\right)\Delta t)\;and\;0<\Delta t<L_{w}.$$

For the whistle with the length of $L_{w}$, the minimum a that distinguishes the whistle slops is calculated by ${\left(\frac{2}{L_{w}}\right)}^{2}$ [20]. Thus, if a is smaller than ${\left(\frac{2}{L_{w}}\right)}^{2}$, the whistle is considered as the zero-slop whistle and Δt is set as $L_{w}$. If a is larger than ${\left(\frac{2}{L_{w}}\right)}^{2}$, Δt is derived from Equation (5). In Equation (5), Δt is the variable of the 2nd order convex function, and the solution of Δt is obtained as $\frac{L_{w}}{2}-\sqrt{\frac{L_{w}{}^{2}}{4}-\frac{1}{a}}\leq \Delta t\leq \frac{L_{w}}{2}+\sqrt{\frac{L_{w}{}^{2}}{4}-\frac{1}{a}}$. Since the minimum Δt is of interest, the orthogonality requirement for the minimum Δt to a and $L_{w}$ is obtained as:

(6) $$\{\begin{array}{c} \Delta t=L_{w},\;a<{\left(\frac{2}{L_{w}}\right)}^{2}\\ \;\frac{L_{w}}{2}-\sqrt{\frac{L_{w}{}^{2}}{4}-\frac{1}{a}}\leq \Delta t,\;{\left(\frac{2}{L_{w}}\right)}^{2}\leq a \end{array}.$$

If Δt dissatisfies Equation (6), the different two whistles are not orthogonal in the time-frequency region. For the orthogonal requirement of the zero-chirp rate whistle, Δt needs to be equal to *L_w_*, which decreases the data rate of the biomimetic TFSK. Note that the whistle duration is several hundred msec to a few seconds.

If the conventional biomimetic TFSK uses few available whistles for the small Δt, the same whistles are frequently re-transmitted, which results in the low DoM. Therefore, this paper proposes the TFSK-based biomimetic modulation using all whistle patterns to increase the DoM.

### 2.2. Proposed Bio-Mimetic TFSK Modulation Method

For the large DoM and the low BER, all types of whistles including the zero-slop whistles need to be utilized and the orthogonal requirements of the short-time unit are crucial.

The proposed method utilizes the sequence with a large autocorrelation to solve the orthogonality problem for the zero-slop-like whistles and the short-time unit problem for the high data rate. The sequence with the large autocorrelation performance is widely used in digital communications to detect the exact time-frequency location when multiple signals exist at the same time [21,22,23,24,25]. If the different good and long sequences are multiplied to the multiple whistles, the time location of each whistle is precisely detected when the multiplied sequence at the receiver is the same as that used in the transmitted whistle. This is because the autocorrelation value of the sequence is large only at the time zero. Thus, if the sequence is utilized in the TFSK, the receiver can detect the exact time location even though Δt is smaller than that in the minimum in Equation (6) and the zero-slop whistles exist.

If the sequence is the vector whose size is 1×L, the sequence (C) is represented as $C=\left[c_{1},\ldots ,c_{l},\ldots ,c_{L}\right]$, where $c_{l}$ denotes the l-th element of the sequence with a value of 1 or −1, and L is the length of the sequence, i.e., cardinality. The ideal C satisfies the following property [21,22,23,24,25]:

(7) $$\sum_{l}c_{l}\times c_{l-n}\;=\{\begin{array}{c} \;\sum_{l}{\left|c_{l}\right|}^{2},\;n=0\\ \;0,\;n\neq 0 \end{array},$$

where $\sum_{l}c_{l}\times c_{l-n}$ denotes the autocorrelation value of the sequence and n denotes a time-lag. Equation (7) is only satisfied when the sequence length (L) is infinite. However, if the sequence with a small L has a good autocorrelation characteristic, the autocorrelation at n≠0 has a very small value which can be considered as zero in Equation (7).

When the sequence C is multiplied to the whistle, the whistle is divided by the cardinality of C and each vector element is sequentially multiplied to the divided whistles. Since the cardinality of C is L, the length ($L_{w}$) of the whistle is divided by L, and a piece (τ) of the whistle is obtained by $L_{w}/L$. The proposed transmission signal multiplied by the sequence is modeled as:

(8) $$S\left(t\right)=\left[\delta \left(t-m\Delta t\right)⊗\left\{c_{t/\tau }\times w\left(t\right)\right\}\right]\times e^{j2\pi n\Delta ft}\;,$$

where A denotes the ceiling function of A. In Equation (8), since τ is inversely proportional to the time resolution, if L increases, the time detection resolution of the whistle also increases, whereas the frequency bandwidth of the whistle is spread out, which distorts the originality of the whistle. Thus, the best L needs to be determined to maximize the time resolution and to minimize the whistle distortion. In this paper, the maximum L is determined by satisfying the undistorted whistle requirement that human does not recognize the distortion of the whistle. The human assessment method is described in Section 3 and Section 4.

Assuming the best length of the sequence is $L_{c}$, the spread frequency ($B_{c}$) of the whistle is calculated as:

(9) $$B_{c}=L_{c}/L_{w}.$$

To satisfy Equation (7), Δt needs to be larger than half of τ, and to satisfy the orthogonality in the frequency domain, Δf needs to be greater than the twice of $B_{c}$. Thus, the time- and orthogonality-requirements of the proposed method are derived as:

(10) $$\{\begin{array}{c} \tau /2\leq \Delta t\\ 2B_{c}\leq \Delta f \end{array}.$$

**Figure 4:**
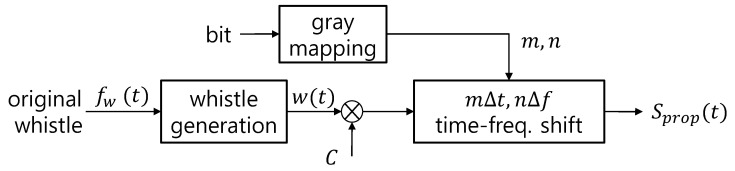
Block diagram of the proposed transmitter.

Note that if the sequence length is large, Δt in Equation (10) is smaller than the minimum value in Equation (6). The block diagram of the proposed transmitter is shown in Figure 4.

For the communication of the proposed method, transmission frames are utilized as in the conventional TFSK [13]. Every frame has a preamble which is made by a whistle with a large chirp rate, and the consecutive whistle patterns and the original time- and frequency-locations of the whistles in the frame are known to the transmitter and the receiver. Thus, the reference information of t=0 and f=0 for every whistle is known, too. What the receiver needs to detect is to estimate the time- and frequency-differences from the reference information.

For the precise detection of the time- and frequency information from the received TFSK whistles, the maximum likelihood (ML) based receiver is proposed. Assume that the whistle is shifted by $m^{*}\Delta t$ in frequency and $n^{*}\Delta f$ in time for an arbitrary input. Then, the modulated whistle ($S_{m^{*},n^{*}}\left(t\right)$) is transmitted. If $S_{m^{*},n^{*}}\left(t\right)$ passes through the underwater acoustic (UWA) channel (h(t)), the received signal (r(t)) is modeled as:

(11) $$r\left(t\right)=h\left(t\right)⊗S_{m^{*},n^{*}}\left(t\right)\;+n\left(t\right),$$

where n(t) denotes AWGN. For the ML detection, the receiver generates the conjugate of the transmitted whistle ($S_{m,n}^{*}\left(t\right))$ for all available time-frequency shifts, in which m and n vary from $-\Delta f\frac{N}{2}$ to $\Delta f\frac{N}{2}$ and $-\Delta t\frac{M}{2}$ to $\Delta t\frac{M}{2}$, respectively. Then, all conjugate whistles are multiplied to the received whistle and each multiplication result is integrated. This procedure is the same as calculating the correlation value (R(m,n)) at n and m. R(m,n) is obtained as:

(12) $$R\left(m,n\right)=\int r\left(t\right)S_{m,n}^{*}\left(t\right)dt.$$

If Δt and Δf satisfy Equation (10), R(m,n) has the largest value at $m=m^{*}$ and $n=n^{*}$, otherwise, R(m,n) has a very small value. Thus, the estimated $\hat{n}$ and $\hat{m}$ of the transmitted indices is calculated by:

(13) $$\left(\hat{m},\hat{n}\right)=\begin{array}{c} \arg\max\\ m,n \end{array}R\left(m,n\right).$$

**Figure 5:**
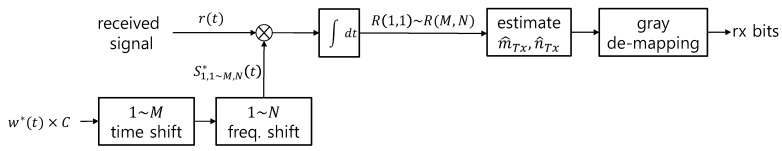
Block diagram of the proposed receiver.

The estimated time-frequency indices using Equation (13) are de-mapped to obtain the transmitted bits. The block diagram of the proposed ML receiver is shown in Figure 5.

The proposed method utilizes the sequence multiplied whistles to transmit any whistle patterns including the zero-slop whistles and increases the DoM. However, the multiplication of the sequence to the whistle causes the frequency spread, and the amount of spread is determined by the sequence length $L_{c}$. In practice, a small amount of frequency spread is unrecognizable to the human. Thus, if a sequence length that generates the unrecognizable frequency spread is chosen, the proposed method can increase the BER without sacrificing the DoM.

The next section describes how to find the unrecognizable sequence length for minimizing Δt, and the DoM assessment for the proposed method.

## 3. Proposed DoM Assessment Method

The DoM is one of the criteria to evaluate the covertness of biomimetic communication. The quantitative and qualitative measurements are proposed to assess the DoM of the biomimetic communication: The quantitative method measures the similarity of the signal shape between one real dolphin and one biomimetic signal using the spectral correlation. The qualitative method measures the similarity of the sound between one real dolphin and one biomimetic signal using a mean opinion score (MOS) test based on human perception. Thus, the qualitative method is more practical than the quantitative method to measure the DoM.

Two conventional measurement methods have been executed for only one whistle, and the DoM of the many consecutive whistles over a long-term duration has not been evaluated. In practice, the dolphins sequentially generate various whistle patterns for the long-time, and the transmitter consecutively transmits many whistles over a long time to transmit many bits. Thus, for the more practical DoM assessment, the consecutive long-term biomimicking whistles need to be tested.

In this paper, a two-step assessment method is performed to measure the DoM of the proposed biomimetic communication. For the 1st step, the various sizes of the sequences are tested to find the sequence length ($L_{c}$) that humans do not recognize. For the 2nd step, the long-term biomimicking whistles using the selected sequence length ($L_{c}$) are evaluated to check whether the size ($L_{c}$) is acceptable for the large DoM. If the evaluation fails, a smaller $L_{c}$ is applied and the 2nd step is executed again. The time of the consecutive whistle is set to 10 s for the 2nd step.

The DoM assessments of each step are based on the MOS test (BS1284) given by the International Telecommunication Union (ITU) [26,27]. The DoM assessment procedure of the 1st step is described in Table 1.

If the MOS score of the modulated whistles by the sequence with L is close to that of the real dolphin whistle, the L is chosen for the sequence length that has unrecognizable frequency spreading. As a result of the 1st step assessment, many Ls are attained. Since the Δf is inversely proportional to the L, the maximum L among the acceptable Ls is selected to maximize the data rate and is set to $L_{c}$.

For the 2nd step, whistles are modulated by the selected $L_{c}$ and the consecutive long-term whistles with a 10-s duration are generated. Then, the 10-s long consecutive long-term whistles are evaluated for the similarity to the real dolphin whistles. The DoM assessment of the 2nd step is described in Table 2.

If the DoM of the proposed method is larger than or close to that of real dolphin whistles, we consider that people may be unable to distinguish the proposed biomimetic consecutive whistles from the real dolphin whistles. Since listeners have no prior information about the test set, the confidence of the evaluation is achieved.

In the next section, the BER performance of the proposed method is analyzed, and $L_{c}$ is selected through the 1st step DoM assessment, and the selected $L_{c}$ is evaluated through the 2nd step DoM assessment.

## 4. Proposed DoM Assessment Method

This section shows that the derived requirement Δt in Equation (10) is correct using computer simulations and that the BERs of the proposed method are better than that in Ref. [13]. Additionally, the 1st step DoM assessment was conducted to find the adequate $L_{c}$, and the 2nd step DoM assessment was executed for the DoM of the long-term signal modulated with the $L_{c}$.

### 4.1. Communication Performance Assessment

For the BER analyses of various whistle patterns, computer simulations were executed under AWGN channel environments. For simplicity, assume that the whistle patterns were modeled as a linear function of at+b. The length ($L_{w}$) of the tested whistles was fixed by 200 ms, the carrier frequency was set as 2 kHz, and four chirp rates (a) of 0 H/s, 1, 2.5, and 5 kHz/s were selected. Then, Δts that satisfy the orthogonality requirements in Equation (6) with the given $L_{w}$ and four chirp rates were calculated. The calculated whistle parameters were displayed in Table 3.

**Figure 6:**
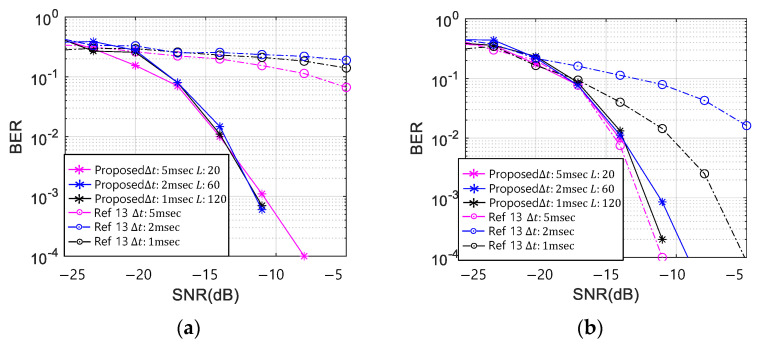

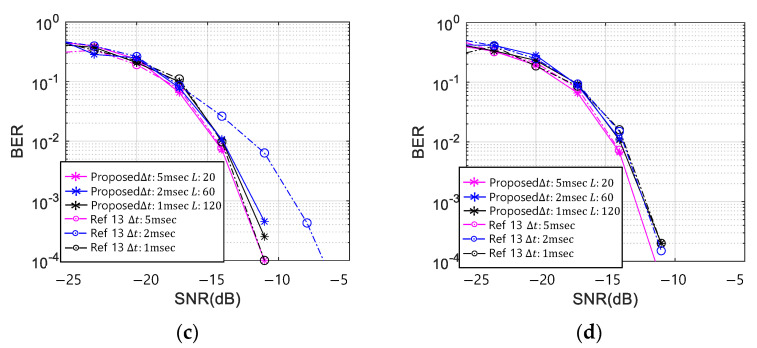
Bit Error Rate (BER) in Additive White Gaussian Noise (AWGN) chirp-rate (a); (**a**) 0 Hz/s, (**b**) 1 kHz/s, (**c**) 2.5 kHz/s, (**d**) 5 kHz/s.

Based on Table 3, the simulation parameters were set. Ls were chosen as 20, 60, and 120, which were also used for the MOS tests. The Kasami sequences for the three sequence lengths were chosen for good autocorrelation performance [28,29,30]. Then, Δts were selected 1, 2, and 5 ms to modulate the whistles. In this simulation, the 200 ms of Δt was excluded because when the Δt exceeded 200 ms, the misdetection by the overlap region did not exist, and the data rate was too low to be utilized as a communication system. The modulation indices of M and N were set as four each, and the total data rate was 20 bits/s. The BER results of the four chirp rate whistles of the proposed scheme and the conventional methods of Ref. [13] with three Δts were shown in Figure 6.

In Figure 6a, for the 0 kHz chirp rate whistle, the BER of the conventional TFSK method did not decrease lower than 0.05, whereas that of the proposed scheme with a good correlation sequence (Kasami) decreases regardless of any Δt. In Figure 6b, for the 1 kHz chirp rate whistle, the conventional TFSK in Ref. [13] showed the poor BER performance for the smaller Δt than 5 ms because the minimum Δt was calculated as 5 ms by Equation (6). In Figure 6c, for the 2.5 kHz chirp rate whistle, the conventional TFSK in Ref. [13] also showed poor BER performance with 1 ms of Δt, which was smaller than 2 ms that was the minimum Δt calculated by Equation (6). However, the BERs of the proposed scheme were not affected for all Δts. This result proved that the BER performance of the conventional TFSK in Ref. [13] depended on both the chirp rate and Δt, whereas the proposed method kept on the good BER performance for any whistle pattern. In Figure 6a–d, when L was 20, the BER performance of the proposed method was good and similar to that of the larger Ls.

### 4.2. Communication Performance Assessment

In this subsection, the DoM experiments were conducted by the proposed assessment procedures of the modulated whistles in Section 3. The 1st step of the proposed assessment was to find $L_{c}$ that provides unrecognizable mimicking and determined the sequence length L. In the 2nd step, the assessment was executed for the DoM with the consecutive long-term whistles based on the obtained L.

**Figure 7:**
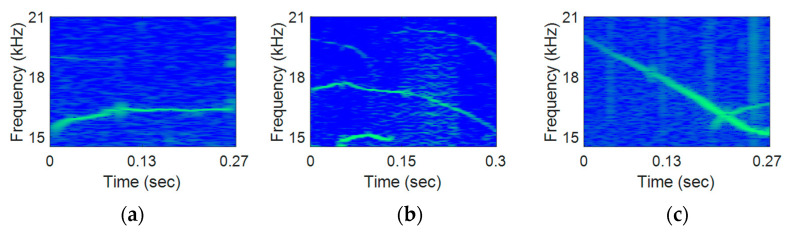

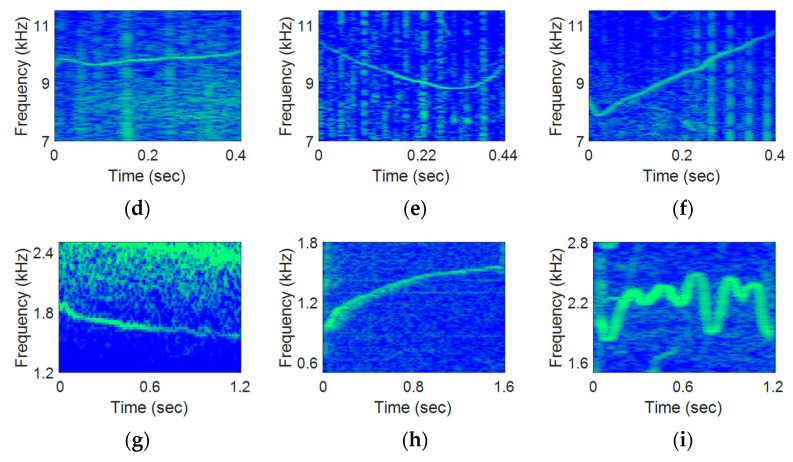
Mean Opinion Score (MOS) test whistle of white sided dolphin of (**a**) flat, (**b**) moderate variation, (**c**) dramatic variation; Delphinus delphis of (**d**) flat, (**e**) moderate variation, (**f**) dramatic variation; Killer whale of (**g**) flat, (**h**) moderate variation, and (**i**) dramatic variation.

For the 1st step DoM assessment, various whistles were chosen from three species, i.e., White-sided dolphin, Delphinus delphi dolphin, Killer dolphin, from the Watkins marine mammal database [31]. Three whistles per species were chosen [31]. The whistle lengths of White-sided dolphin, Delphinus delphi dolphin, and Killer dolphin were about 0.3, 0.4, and 1.3 s, respectively. The spectrograms of the selected whistles are shown in Figure 7.

For the 1st step DoM assessment, the sequences with Ls of 20, 60, 100, and 120 were multiplied to the whistles for the modulation. A set of assessment whistles consisted of one of the original dolphin whistles in Figure 7 and four biomimetic whistles that were modulated from the real dolphin whistles with four Ls. Noises were added to the real- and the modulated-whistles with 10 and 20 dB of SNRs to analyze the noise effects to the DoM assessments. The number of listeners was 30. The ages of the listeners were from 10 to 60 s, and the age of listeners are uniformly distributed. The 1st step DoM assessment was conducted in Table 1. The listeners graded the similarity to the real dolphin whistles by Table 4.

For the assessment test, Terratec D/A convertor and AKG-K52 headphone were utilized. The assessment results are shown in Table 5.

In Table 5, as the SNR decreased, the average MOS of the modulated whistles increased. This is because the background noise hindered distinguishing the difference between the modulated- and the original-whistles. The MOSs of real Delphinus delphis and White-sided dolphin for all sequence lengths were approximately 4.2. As a result of the 1st step assessment, the human does not recognize the difference between real whistle and whistle multiplied with the sequence length of 120. Thus, the maximum sequence length for Delphinus delphis and White-sided is 120, and the spreading bandwidth ($B_{c}$) was calculated as 300 Hz using Equation (9). The MOS score of a real killer whale was higher than whistles multiplied with the sequence. If we used a smaller sequence length, the MOS score of sequence multiplied whistle increased. In this paper, Delphinus delphis was selected as a mimetic model because the MOSs of Delphinus delphis’s real whistle and sequence multiplied whistle were the same. Based on this assessment, the largest length of the sequence by the 1st step DoM assessment was selected as 120 and $B_{c}$ was chosen as 300 Hz.

**Figure 8:**
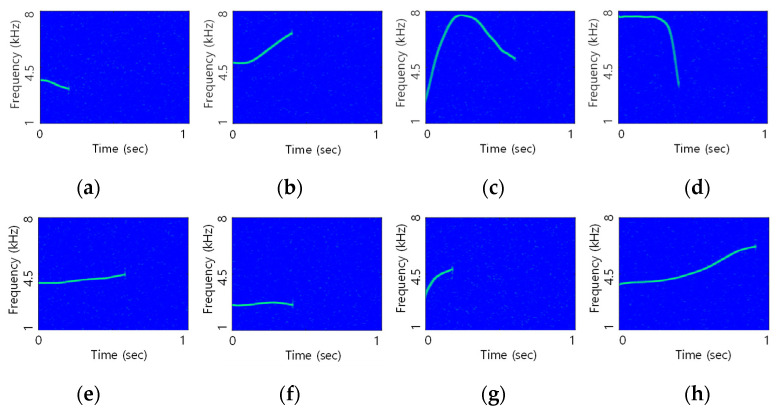
Whistle for the 2nd step DoM assessment (**a**) moderate change, (**b**) moderate change, (**c**) dramatic change, (**d**) dramatic change, (**e**) flat, (**f**) flat, (**g**) dramatic change, (**h**) moderate change.

The 2nd step DoM assessment was conducted using the result of the 1st step. For the 2nd step DoM assessment, eight Delphinus delphis dolphin whistles were modulated by TFSK and each whistle was multiplied by the sequence that has 300 Hz of $B_{c}$. The spectrograms of the modulated whistles are shown in Figure 8.

Two consecutive long-term whistle signals with a 10-s duration were made: the first signal consisted of eight whistles in Figure 8. The second one was composed of two whistles in Figure 8c,g. The first and the second signals were utilized for the MOS tests of the proposed method and the conventional method, respectively. For the fair MOS comparison tests, the BERs of the two methods needed to be the same. Since Δt was set as 5 ms, the whistles that satisfied the Δt in Equation (6) were (c) and (g) in Figure 8.

The test sets consisted of the two generated whistles and a 10-s long real dolphin whistle. Listeners graded how close the test set sounds were to the real dolphin whistle sound by Table 3. The listeners and equipment used in the 2nd assessment were the same as in the first experiment. The 2nd assessment results are shown in Table 6.

In Table 6, the MOS of the proposed method was 3.81, and the MOS of the real dolphin was 3.72. This result meant humans were unable to distinguish the modulated whistles by the proposed method from the real dolphin whistles. However, the MOS of the conventional method in Ref. [13] was 2.81, which was lower than that of the real dolphin whistles. In other words, humans recognized the modulated whistles by the conventional one. This was because repeatedly transmitted whistles enabled humans to recognize the difference between the artificial whistles and the real ones.

The MOS test demonstrated that humans could not distinguish the long-term consecutive whistles by the proposed method from the real dolphin whistle sound. Therefore, the proposed method achieved the large DoM that was one of two goals, i.e., large DoM and low BER, of the covert communications. In the next section, the other goal, i.e., low BER, is demonstrated through the computer simulation with the UWA channels and the practical lake and ocean experiments.

## 5. Simulations and Experiments

The BER comparison of the proposed method and the conventional one was executed by the simulation and practical lake and ocean experiments. For the fair BER comparisons, the DoMs of the two methods needed to be the same, and all whistles in Figure 8 needed to be utilized. The TFSK modulation parameters of M and N were four each and $B_{c}$ was chosen as 300 Hz as in Section 4. Since the shortest whistle time in Figure 8 was measured by 0.13 s, the minimum sequence length was set as 39 by Equation (9) and the Kasami sequence with the length of 39 was used for the proposed method. When the sequence length was 39, Δt was chosen as 5 ms and Δf was set to 600 Hz by Equation (10). The modulation parameters of M, N, Δt, and Δf were the same for the proposed method and the conventional one.

### 5.1. Simulation Experiments

**Figure 9:**
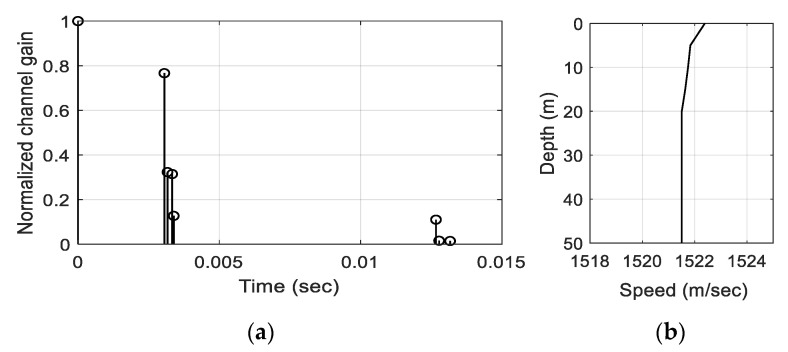
Simulation channel (**a**) delay profile, (**b**) sound velocity profile (SVP).

The UWA environments were modeled from a point of Taean in the West Sea of S. Korea, and the UWA channel was modeled by Bellhop based on the environments. The doppler spread was set as 2 Hz, which was measured at the same location [32,33]. The delay profile and sound velocity profile (SVP) of the UWA channel are shown in Figure 9.

**Figure 10:**
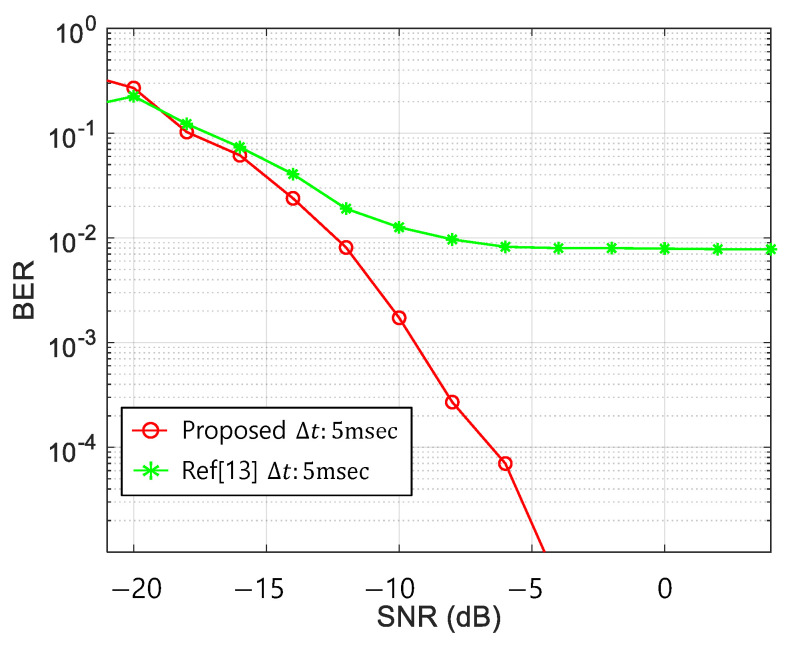
BER result in Under Water Acoustic (UWA) channel.

The BER obtained by the UWA channel in Figure 9 is shown in Figure 10.

In Figure 10, the red- and green-line denote the BERs of the proposed- and the conventional-methods, respectively. When the conventional TFSK signals passed through the multipath channels, the time spreading occurred, which caused the detection ambiguity for the zero-chirp rate whistles. Thus, the BER of the conventional method had an error floor at 10^−2^, even though the SNR is large, e.g., SNR > −10 dB. However, the BER of the proposed method did not have the error floor at Δt of 5 ms which satisfies the orthogonality requirement by Equation (7) and showed less than 10^−4^ at a −5 dB SNR, which was an acceptable value for the practical communications.

### 5.2. Lake and Ocean Experiments

The lake and ocean experiments were conducted to verify the BER performance of the proposed method and the conventional one. The communication parameters in the lake and ocean experiment were the same as in the computer simulations.

**Figure 11:**
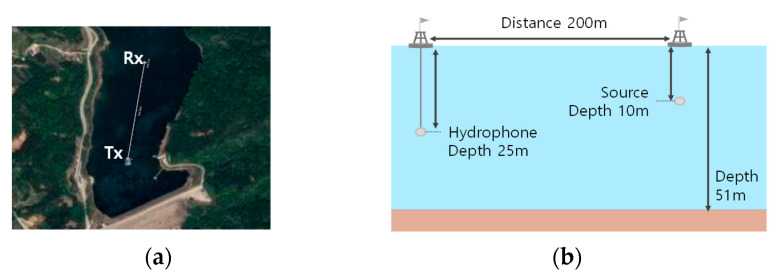

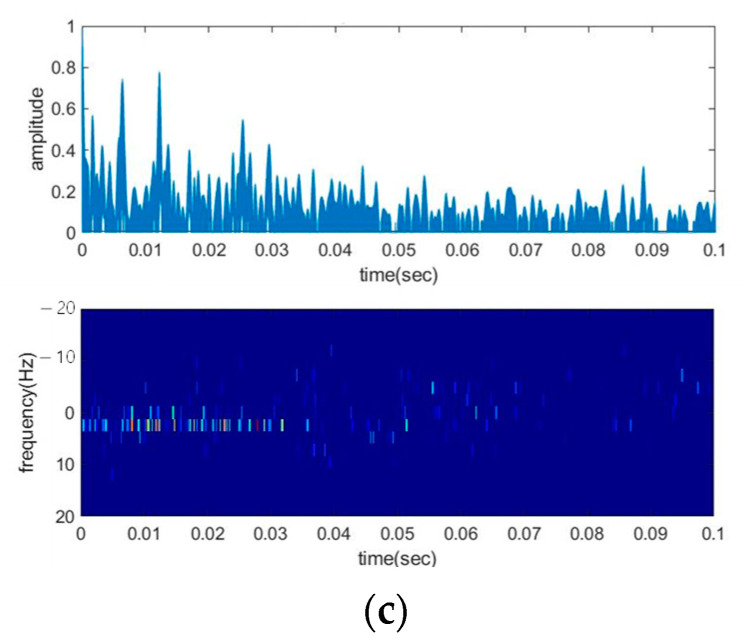
Lake experiments environments (**a**) Location, (**b**) Configuration, (**c**) delay profile, and doppler spread.

The lake experiments were executed at Lake Kyungchun on 13 May 2020. The transmitter was deployed at a depth of 10 m from the surface, and a Neptune-D17BB with a frequency band from 12.5 to 19.5 kHz was used. Since the available frequency band of the transmitter was greater than that of the real dolphin whistles, the real dolphin whistles were shifted up to the available frequency band of the transmitter. Note that the frequency-shifted whistle patterns were the same as the real dolphin whistle ones. TC4032 was used for the hydrophone at a 25 m depth. The distance between transmitter and receiver was 200 m. In Figure 11, the location, configurations, measured delay profile, and doppler spread were displayed.

In Figure 11c, the rms delay and the doppler spread were measured as 100 ms, 2 Hz, respectively.

**Figure 12:**
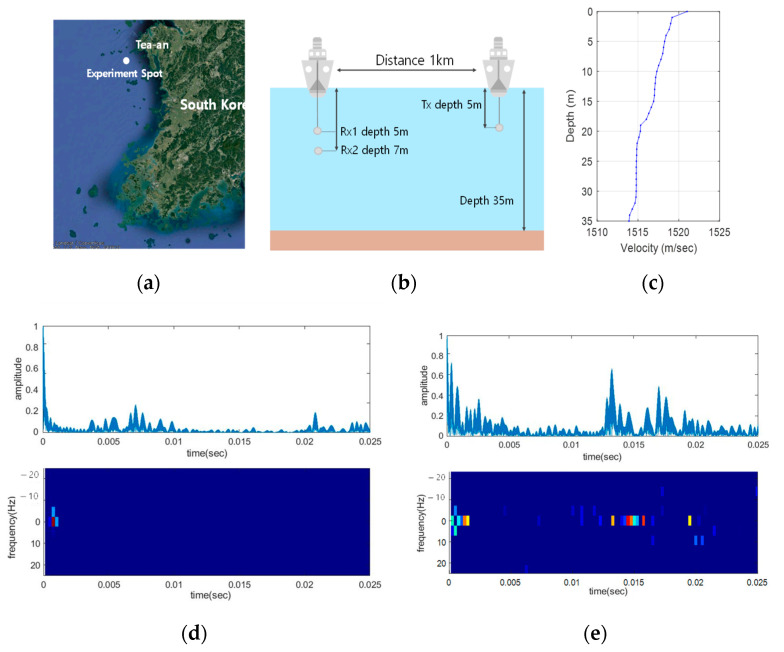
Ocean experiments environments:(**a**) Location, (**b**) Configuration, (**c**) SVP, Delay profile and doppler spread: (**d**) 12:30 p.m., (**e**) 5:44 p.m.

The ocean experiments were performed on 13 September 2020. The experiment spot was 7 km far away from the coast of Sinjindo, Taean-gun, S. Korea. The equipment used in the ocean experiments was the same as in the lake experiments. The transmitter was deployed at a depth of 5 m from the sea level, Two-channels of TC4032 were used for the receiver at 5 m and 7 m depths. The distance between transmitter and receiver was 1 km. Figure 12 from (a) to (e) showed the experiment location, the configurations of the experiments, SVP, and the estimated delay profile and doppler spread, respectively.

In Figure 12d,e, the estimated delay profiles and the doppler spread were demonstrated at 12:30 p.m. and 5:44 p.m., respectively. The same doppler spread were calculated as 2 Hz for two cases. Even though the parameters were measured at the same location, these UWA channels were different because of the large tide difference.

**Figure 13:**
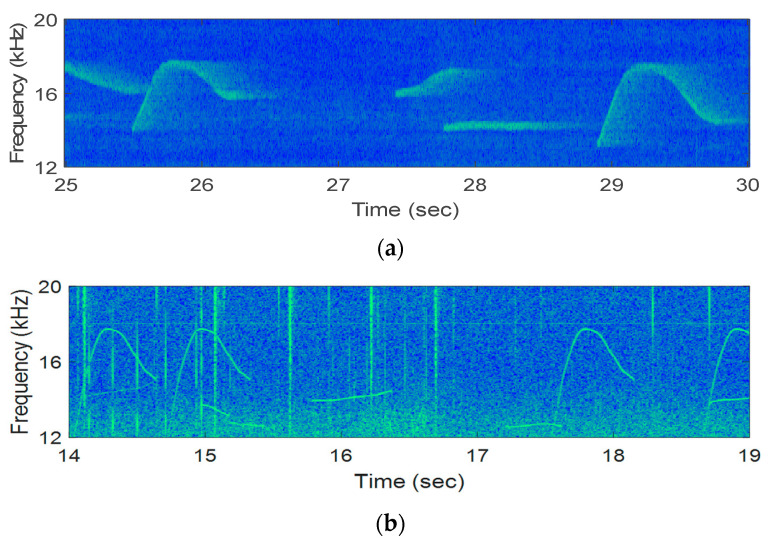
Experiments received signals: (**a**) Ocean, (**b**) Lake.

Figure 13 exhibited the examples of the received signals of the lake and the ocean experiments. The number of transmitted bits for the lake and ocean experiments were 4000 and 10,000, respectively.

The BERs obtained from the lake and the ocean experiments were shown in Table 7. The proposed method demonstrated a zero-error rate for two experiments, whereas the conventional method exhibited 0.14 and 0.24 for the lake and the ocean experiments, respectively. In ocean experiments, the SNR of the received signal was estimated as −6 dB. Since the zero error among 10,000 bits was found, the BER was expected to less than 10^−4^, which was well matched with the BER by the computer simulation results in Figure 10. However, at the same SNR, the BER of the conventional method was 0.24 which was large and was not acceptable to practical communications.

## 6. Conclusions

This paper proposes a biomimicking modulation method with the large DoM and the low BER. The proposed method utilizes the sequence with a large correlation characteristic to enhance the conventional TFSK and develops the ML detector for the transmitted signal and derives the orthogonality requirements for the time- and frequency-shift units. The proposed method also develops a two-step MOS assessment for evaluating the DoM for the long-term whistle signal. The 1st step assessment determines the minimum length of the sequence and the 2nd step confirms the selected length of the sequence for the consecutive long-term whistles. By the assessments, the fact that the modulated whistles by the proposed scheme cannot be distinguished from that by the real dolphin sound is shown. Computer simulations demonstrate that the BER performance of the proposed method is better than that of the conventional one, and the practical lake and ocean experiments demonstrate zero error when 4000 bits and 10,000 bits were transmitted, respectively.

## Figures and Tables

**Table 1:** The 1st step Degree of Mimic (DoM) assessment.

Modulate the whistles with various sequence lengths (*L*) for the test sets Prepare the test sets of the modulated whistles and a real dolphin whistle Firstly, listeners listen to the real dolphin whistle Then, listeners sequentially listen to the randomly selected whistles among the test set including the real dolphin whistle. Note that the real dolphin whistle is also evaluated for the reference assessment Listeners grade the amount of similarity for all tested whistles by how close the test whistle is to the real dolphin whistle according to the MOS grade score. The DoM for a specific sequence length (*L*) is obtained by averaging MOS scores of the modulated whistles.

**Table 2:** The 2nd step DoM assessment.

Generate 10-s long consecutive whistles using the modulated whistles with *L_c_* Make the test set using the modulated whistles and 10-s long real dolphin whistles Listeners listen to the signals of the test set in random order Listeners evaluate how close the test whistles are to the real dolphin whistles according to the MOS grade score The DoM for a specific test signal is obtained by averaging MOS scores.

**Table 3:** Whistle parameters.

Parameter	Values			
b (kHz)	2			
a (kHz/s)	0	1	2.5	5
Δt (ms)	200 ≤Δt	5 ≤Δt	2 ≤Δt	1 ≤Δt

**Table 4:** MOS grades for the 1st step DoM assessment.

1	2	3	4	5
Different	Slightly different	Similar	Very similar	same

**Table 5:** Results of the 1st step DoM assessment.

Species	Noise	Real Dolphin	Sequence Length (L)			
			20	60	100	120
Killer	10 dB	4.7	3.8	3.2	3.1	3.2
	20 dB	4.8	3.9	3.0	2.9	3.1
Delphinus delphis	10 dB	4.1	4.3	4.3	4.2	4.3
	20 dB	4.2	4.3	4.0	4.1	4.1
Whistle sided	10 dB	4.2	4.1	4.1	4.2	4.1
	20 dB	4.4	4.2	4.2	4.2	4.2

**Table 6:** Average MOS result for the 2nd assessment.

Real Dolphin	Proposed Method	Conventional Method (Ref. [13])
3.72	3.81	2.81

**Table 7:** BER results of the lake and the ocean experiments.

Location	Demodulation Scheme	BER
Lake	Proposed method	0.00
	Conventional method (Ref. [13])	0.14
Ocean	Proposed method	0.00
	Conventional method (Ref. [13])	0.24

## Data Availability

Not applicable.
